# Supplementary material for: The Influence of an Extended π Electron System on the Electrochemical Properties and Oxidizing Activity of a Series of Iron(III) Porphyrazines with Bulky Pyrrolyl Substituents
Source: Molecules. 2023 Oct 22;28(20):7214. doi: 10.3390/molecules28207214 (PMC10609377; doi:10.3390/molecules28207214)
Supplement: Supplementary file 1 [file molecules-28-07214-s001.zip › molecules-2653575-supplementary.pdf]

SUPPLEMENTARY MATERIALS

The Influence of Extended π Electron System on the Electrochemical Properties and Catalytic Activity of a Series of Iron(III) Porphyrazines with Bulky Pyrrolyl Substituents

Tomasz Koczorowski [1,*] and Tomasz Rębiś [2]

[1] Chair and Department of Chemical Technology of Drugs, Poznan University of Medical Sciences, Rokietnicka 3, 60-806 Poznan, Poland; e-mail: tkoczorowski@ump.edu.pl

[2] Institute of Chemistry and Technical Electrochemistry, Poznan University of Technology, Berdychowo 4, 60-965 Poznan, Poland; e-mail: tomasz.rebis@put.poznan.pl

[*]Correspondence: e-mail: tkoczorowski@ump.edu.pl (T.K.)

[2,7,12,17-Tetrakis(dimethylamino)-3,8,13,18-tetrakis-(2,5-dimethyl-1H-pyrrol-1-yl)porphyrazinato]iron(III) chloride (**Pz1**): dark green solid (41 mg, 46% yield). m.p. > 300 °C. R_f (CH_2Cl_2:CH_3OH, 50:1) 0.29. UV-Vis (CH_2Cl_2): λ_{max}, nm (log ε) 310 (4.75), 373 (4.53), 429 (4.53), 552 (4.41), 685 (4.70), 916 (4.26). ^{1}H NMR (500 MHz; pyridine-d_5): δ_H, ppm 1.99 (s, 24H, pyrrolyl-CH_3), 2.20–2.33 * (m, pyrrolyl-CH_3), 2.74 (s, 24H, $N(CH_3)_2$), 3.39–3.50 * (m, $N(CH_3)_2$), 5.85 (s, 8H, pyrrolyl-H), 6.27–6.34 * (m, pyrrolyl-H). ^{13}C NMR (125 MHz; pyridine-d_5): δ_C, ppm 13.4, 14.4 *, 40.2 ($N(CH_3)_2$), 42.8 * ($N(CH_3)_2$), 107.0 *, 107.8, 132.3, 139.4, 151.8, 152.8, 165.2. An asterisk (*) indicates the aggregated specie. HRMS (MALDI TOF): *m/z* Calc. for $C_{48}H_{56}N_{16}Fe[M]^+$ 912.4223, found 912.6889. HPLC purity 94.58–100%.

[2,7,12,17-Tetrakis(dimethylamino)-3,8,13,18-tetrakis-(2-methyl-5-phenyl-1H-pyrrol-1-yl)porphyrazinato]iron(III) chloride (**Pz2**): dark green solid (27 mg, 54% yield). m.p. > 300 °C. R_f (CH_2Cl_2:CH_3OH, 50:1) 0.26. UV-Vis (CH_2Cl_2): λ_{max}, nm (log ε) 289 (4.97), 377 (4.44), 434 (4.45), 553 (4.32), 689 (4.60), 917 (4.21). ^{1}H NMR (500 MHz; pyridine-d_5): δ_H, ppm 2.10–2.78, 3.25–3.78 (2×m, 36H, pyrrolyl-CH_3 and $N(CH_3)_2$), 6.57–6.93 (m, 20H, C_6H_5), 7.58–7.65 (m, 8H, C_6H_5). ^{13}C NMR (125 MHz; pyridine-d_5): δ_C, ppm 13.1, 14.1, 14.3, 39.0, 39.9, 42.5, 108.2, 109.0, 109.1, 109.3, 110.3, 126.3, 127.0, 127.5, 127.7, 128.0, 128.8, 128.9, 129.6, 129.9, 135.7, 136.5, 138.1, 149.9. HRMS (MALDI TOF): *m/z* Calc. for $C_{68}H_{64}N_{16}Fe$ $[M]^+$ 1160.4849, found 1160.6684. HPLC purity 97.97–98.57%.

[2,7,12,17-Tetrakis(dimethylamino)-3,8,13,18-tetrakis-(2,5-diphenyl-1H-pyrrol-1-yl)-porphyrazinato]iron(III) chloride (**Pz3**): dark green solid (20 mg, 22% yield): mp > 300ºC. Rf (CH_2Cl_2:CH_3OH, 50:1) 0.17. UV-Vis (CH_2Cl_2): λmax, nm (logε) 296 (4.58), 381 (3.91), 440 (3.90), 691 (3.90), 911 (3.56). ^{1}H NMR (400 MHz; pyridine-d_5): δH, ppm 2.69 (s, 24H, $N(CH_3)_2$), 2.91*-3.20* (m, 24H, $N(CH_3)_2$), 6.58 (s, 8H, pyrrole-H), 6.74* (t, ^{3}J = 6 Hz, 24H, C_6H_5), 7.00* (s, 8H, pyrrole-H), 7.30 (t, ^3J = 6 Hz 24H, C_6H_5), 7.50* (d, ^3J = 3 Hz, 16H, C_6H_5), 7.67 (d, ^3J = 3 Hz, 16H, C_6H_5). ^{13}C NMR (175 MHz; pyridine-d_5): δC, ppm 39.98 ($N(CH_3)_2$), (42.26*-42.63*) ($N(CH_3)_2$), (110.54*), 111.73, 111.73, (128.20*), 128.44, 128.44, (128.44*), 128.82, 134.17, (134.17*), 135.20, 140.37, 140.37, (140.37*), 145.25. An asterisk (*) indicates the aggregated specie. MS (MALDI TOF): m/z 1408.6 $[M+H]^+$.

[2,7,12,17-Tetrakis(dimethylamino)-3,8,13,18-tetrakis-(2,3,5-triphenyl-1H-pyrrol-1-yl)porphyrazinato]iron(III) chloride (**Pz4**): dark green solid (15 mg, 58% yield). m.p. > 300 °C. R_f (CH_2Cl_2:CH_3OH, 50:1) 0.33. UV-Vis (CH_2Cl_2): λ_{max}, nm (log ε) 298 (5.24), 378 (4.59),

436 (4.58), 547 (4.41), 692 (4.69), 915 (4.37). ^{1}H NMR (500 MHz; pyridine-d_5): δ_H, ppm 2.83–3.69 (m, 24H, $N(CH_3)_2$), 6.54–7.07 (m, 26H, C_6H_5), 7.15–7.16 (d, 1H, C_6H_5), 7.24–7.26 (m, 6H, C_6H_5), 7.30–7.40 (m, 15H, C_6H_5), 7.45–7.50 (m, 4H, pyrrolyl-H), 7.61 (s, 1H, C_6H_5), 7.67–7.71 (m, 9H, C_6H_5), 7.77–7.78 (m, 2H, C_6H_5). ^{13}C NMR (125 MHz; pyridine-d_5): δ_C, ppm 39.5, 41.8, 110.0, 110.1, 110.2, 124.3, 125.9, 126.2, 126.3, 127.0, 127.1, 127.7, 127.8, 127.9, 128.0, 128.2, 128.3, 128.5, 129.0, 129.2, 131.1, 131.3, 131.4, 131.5, 132.4, 132.4, 133.8, 133.9, 134.3, 134.5, 134.6, 137.6, 137.7, 137.8, 145.5, 147.2, 149.4. HRMS (MALDI TOF): *m*/*z* Calc. for $C_{112}H_{89}N_{16}Fe[M + H]^+$ 1713.6806, found 1713.1149. HPLC purity 95.07–96.51%.
